# A Hybrid Machine Learning and Network Analysis Approach Reveals Two Parkinson’s Disease Subtypes from 115 RNA-Seq Post-Mortem Brain Samples

**DOI:** 10.3390/ijms23052557

**Published:** 2022-02-25

**Authors:** Andrea Termine, Carlo Fabrizio, Claudia Strafella, Valerio Caputo, Laura Petrosini, Carlo Caltagirone, Raffaella Cascella, Emiliano Giardina

**Affiliations:** 1 Data Science Unit, IRCCS Santa Lucia Foundation c/o CERC, 00143 Rome, Italy; a.termine@hsantalucia.it (A.T.); c.fabrizio@hsantalucia.it (C.F.); 2 Genomic Medicine Laboratory UILDM, IRCCS Santa Lucia Foundation, 00179 Rome, Italy; claudia.strafella@gmail.com (C.S.); v.caputo91@gmail.com (V.C.); 3 Medical Genetics Laboratory, Department of Biomedicine and Prevention, Tor Vergata University, 00133 Rome, Italy; raffaella.cascella@gmail.com; 4 Experimental and Behavioral Neurophysiology, IRCCS Santa Lucia Foundation c/o CERC, 00143 Rome, Italy; laura.petrosini@uniroma1.it; 5 Department of Clinical and Behavioral Neurology, IRCCS Santa Lucia Foundation, 00179 Rome, Italy; c.caltagirone@hsantalucia.it; 6 Department of Biomedical Sciences, Catholic University Our Lady of Good Counsel, 1000 Tirana, Albania; 7 UILDM Lazio ONLUS Foundation, Department of Biomedicine and Prevention, Tor Vergata University, 00133 Rome, Italy

**Keywords:** data science, genomic data science, machine learning, network analysis, RNA-seq, precision medicine, subtyping, Parkinson’s disease

## Abstract

Precision medicine emphasizes fine-grained diagnostics, taking individual variability into account to enhance treatment effectiveness. Parkinson’s disease (PD) heterogeneity among individuals proves the existence of disease subtypes, so subgrouping patients is vital for better understanding disease mechanisms and designing precise treatment. The purpose of this study was to identify PD subtypes using RNA-Seq data in a combined pipeline including unsupervised machine learning, bioinformatics, and network analysis. Two hundred and ten post mortem brain RNA-Seq samples from PD (*n* = 115) and normal controls (NCs, *n* = 95) were obtained with systematic data retrieval following PRISMA statements and a fully data-driven clustering pipeline was performed to identify PD subtypes. Bioinformatics and network analyses were performed to characterize the disease mechanisms of the identified PD subtypes and to identify target genes for drug repurposing. Two PD clusters were identified and 42 DEGs were found (*p* adjusted ≤ 0.01). PD clusters had significantly different gene network structures (*p* < 0.0001) and phenotype-specific disease mechanisms, highlighting the differential involvement of the Wnt/β-catenin pathway regulating adult neurogenesis. *NEUROD1* was identified as a key regulator of gene networks and ISX9 and PD98059 were identified as *NEUROD1*-interacting compounds with disease-modifying potential, reducing the effects of dopaminergic neurodegeneration. This hybrid data analysis approach could enable precision medicine applications by providing insights for the identification and characterization of pathological subtypes. This workflow has proven useful on PD brain RNA-Seq, but its application to other neurodegenerative diseases is encouraged.

## 1. Introduction

Parkinson’s disease (PD) is the most common age-related motor neurodegenerative disease, affecting more than 6 million people worldwide, with rising incidence and prevalence imposing a mounting socioeconomic burden on society [1,2,3] and currently, no disease-modifying treatments are available [4,5,6]. The genetic basis of PD has been evaluated with several genome-wide association studies (GWASs), finally reporting up to 90 PD-associated risk variants in various cohorts [7,8,9,10]. However, genetic variants account for ~20% of PD familial cases, while the etiology of most idiopathic cases is largely unclear as multiple genes and environmental factors are believed to be involved in idiopathic PD onset and progression [7]. Consequently, PD is heterogeneous in both clinical manifestations and progression, which serves as evidence for the existence of disease subtypes [11,12]. Defining which PD subtype we are facing is crucial to better understand underlying mechanisms, predict disease course, and eventually design personalized management strategies able to fully consider the genetic or other specific biological features that can be employed in a precision medicine approach addressed to match the patients’ needs [11,13,14,15].

Empirical clustering stratifies patients based on demographic factors, clinical parameters, and genetic factors, making use of expert-based a priori conceptions. Up to now, these applications have shown limited sensitivity in detecting clinically useful classes of PD patients, thus hindering the development and deployment of better suited treatments [16]. It has been shown that the use of a priori assumptions in stratifying patients with complex diseases like PD can be appealing due to its simplicity; nevertheless, this method has an arbitrary nature [16]. As an example, PD patients younger than 50 years of age represent only 5–10% of the total population [17] and thus they are simple to subset, whereas the later onset subtype (~90%) remains highly heterogeneous in its clinical presentation, and a clear boundary between groups is not easily achieved. The unreliability of this subtyping method became apparent as many studies used arbitrary cut-offs of 50, 55, and 60 [16,18]. Similarly, various authors have defined the motor subtypes of PD differently, providing ambiguous results for patient stratification, which indicates the arbitrary nature of a priori conceptions [16]. As a fine-grained diagnosis is pivotal for precision medicine applications, more sensitive methods are required. To this extent, data-driven clustering based on unsupervised machine learning (ML) could offer better results by applying unbiased statistical methods and a hypothesis-free approach. In fact, clustering algorithms aim at finding patterns within data distribution to define clusters, free from a priori assumptions about disease and patients. Data-driven subtyping applications on complex diseases are shaping our knowledge about the best suited treatments for patients with a fine-grained diagnostic definition, such as molecular subtypes of Alzheimer’s disease or colorectal cancer [19,20]. Coupling data-driven clustering methods with next-generation sequencing (NGS) is advancing biomedical research, and transcriptomics data from RNA-Seq can be used in gene-network analysis to identify gene co-expression/co-regulation patterns, refining our understanding of complex biological systems. Data-driven PD subtyping has so far provided heterogeneous results due to disparate data sources and methods of clustering, which hampers understanding of idiopathic PD subgrouping [16]. In fact, even if data-driven clustering is a hypothesis-free approach, its results are dependent on the choice of the number of clusters and the clustering technique used. A fully data-driven clustering pipeline would address these limitations, providing more detailed diagnoses to facilitate precision medicine applications.

To the best of our knowledge, there are no published attempts at molecular subtyping of PD. Transcriptomic profiling from RNA-Seq data provides an in-depth characterization of complex diseases affecting the brain, reflecting the heterogeneity in the biological profiles of such pathologies. Moreover, network biology has not been extensively applied to PD research due to the lack of available data, thus information about gene interactions and regulations in PD is still poor [21]. In addition, there are no available disease-modifying treatments for PD. Disease heterogeneity can be tidied up by coupling unsupervised ML methods with bioinformatics and network analyses on transcriptomic profiles. Regulatory network identification would highlight genes acting as key regulators in each subtype, which can be used in a drug repurposing pipeline to foster tailored treatment definition. Here we show the implementation of a fully data-driven clustering pipeline and its application to PD subtyping. Our proposed novel methodology for better detailing diagnosis and accelerating drug repurposing could provide new research insights for assessing the efficacy of treatments. Our hybrid data-driven workflow is applied to gene expression data from idiopathic PD post-mortem brain samples from Gene Expression Omnibus (GEO) and PubMed databases. We aim to demonstrate that coupling unsupervised ML for disease subtyping with bioinformatics and network analysis for regulatory network identification can clarify the molecular landscape of disease subtypes. The workflow integrates multiple unsupervised ML algorithms for disease subtyping, with independent clusterability assessment and best number of clusters determination. The clustering pipeline aims to optimize the internal clustering validation measures. Network and bioinformatics analyses have been used to isolate and characterize regulatory genes in common and specific disease networks. The obtained key genes from this workflow were tested in a drug repurposing pipeline, enabling us to propose a set of compounds with disease-modifying potential.

## 2. Results

### 2.1. Clustering Results and Clinical Characterization

**Figure 1:**
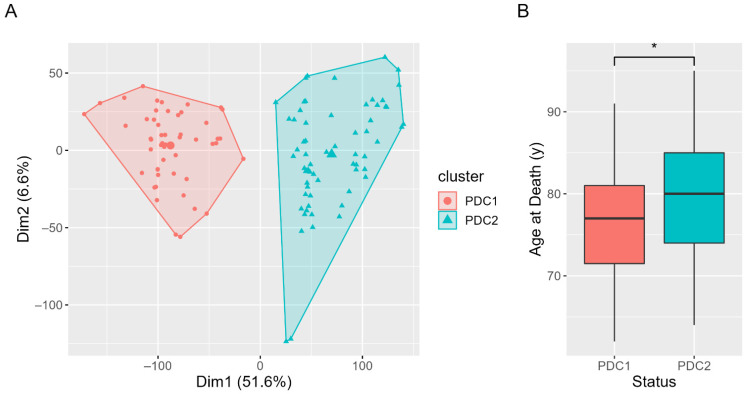
Clustered subjects and clinical characterization. (**A**) PCA plot for RNA-Seq data, clearly showing PDC1 and PDC2 separation. (**B**) Boxplot for PDC1 and PDC2 age at death, reporting a significant difference * *p* value < 0.05.

Two subtypes of PD, namely PD Cluster 1 (PDC1, *n* = 50) and PD Cluster 2 (PDC2, *n* = 65), were identified through the cluster analysis pipeline on gene-expression data. Hierarchical K-means implementing the centroid method with Manhattan distance was selected as the best clustering algorithm based on validation metrics (Silhouette_avg_ = 0.40; Dunn = 0.05). The differences between PDC1 and PDC2 clinical and demographic variables were assessed. PDC2 lives longer than PDC1 (Wilcoxon Test *p* value = 0.03), and lower Braak scores than PDC1 were found (Fisher Test *p* value = 0.004), while no sex differences in the composition of the clusters were found (Fisher Test *p* value = 0.21) (Figure 1).

### 2.2. Differentially Expressed Genes in PDC1 vs. PDC2

**Figure 2:**
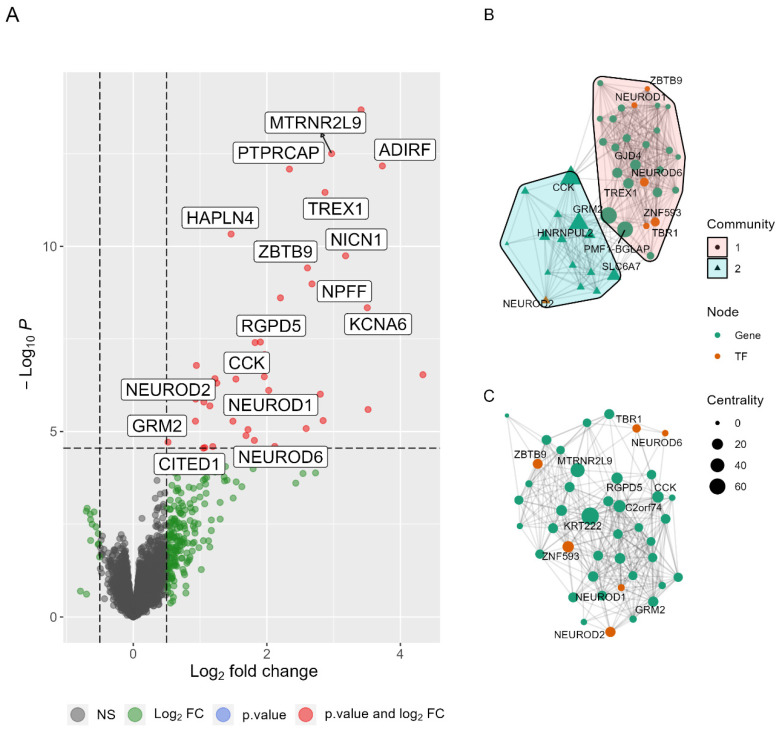
Differences between PDC1 and PDC2 gene expression. (**A**) Volcano plot reporting differences in gene expression where red points were DEGs. (**B**) Gene network for PDC1 showing nodes are segregated into 2 communities. (**C**) Gene network for PDC2, showing no segregation in communities. Both networks were built with the Kamada–Kawai layout, where each node is a gene and each edge is a PCIT value between genes.

To investigate the genetic background regulating the differences in the survival rate of the two PD clusters, we performed a differential gene expression analysis to retrieve DEGs. A comparison between the gene expression profiles of PDC1 and PDC2 was performed, yielding 42 DEGs with *p* adjusted ≤ 0.01 (Figure 2A). Among them, 6 transcription factors (TFs) were identified, mostly regulating the Wnt/β-catenin pathway including *TBR1*, the basic helix–loop–helix (bHLH) TF members *NEUROD1*, *NEUROD2*, *NEUROD6*, coupled with CBP/p300 modulators (*CITED1*, *eEF1Bγ*). Notably, only 6% of the DEGs were recognized as PD-associated genes by GDA analysis. Functional enrichment analysis revealed that DEGs were mostly enriched in protein complexes associated with CBP/p300 modulation in the Wnt/β-catenin pathway, namely IRF3-CBP (CORUM:299 *p* adjusted < 0.01), BETA2-Cyclin D1 (CORUM:2635 *p* adjusted < 0.01), SNW1 (CORUM:298 *p* adjusted < 0.05), and VEGF (CORUM:298 *p* adjusted < 0.05) complexes. Regulation of drug responses (GO:2001023 *p* adjusted < 0.05) was also altered between PDC1 and PDC2, with *ADIRF* and *GRM2* overexpressed in PDC2. Overall, the obtained DEGs differently modulated the regulation of synapse structure or activity (GO:0050803 *p* adjusted < 0.05; GO:0006865 *p* adjusted < 0.05), including alterations in the glutamatergic synapse due to mGluR2-mGluR4 complex (CORUM:6363 *p* adjusted < 0.01) modulation.

### 2.3. PDC1 and PDC2 Networks Differ for Neuroprotective Pathways

**Figure 3:**
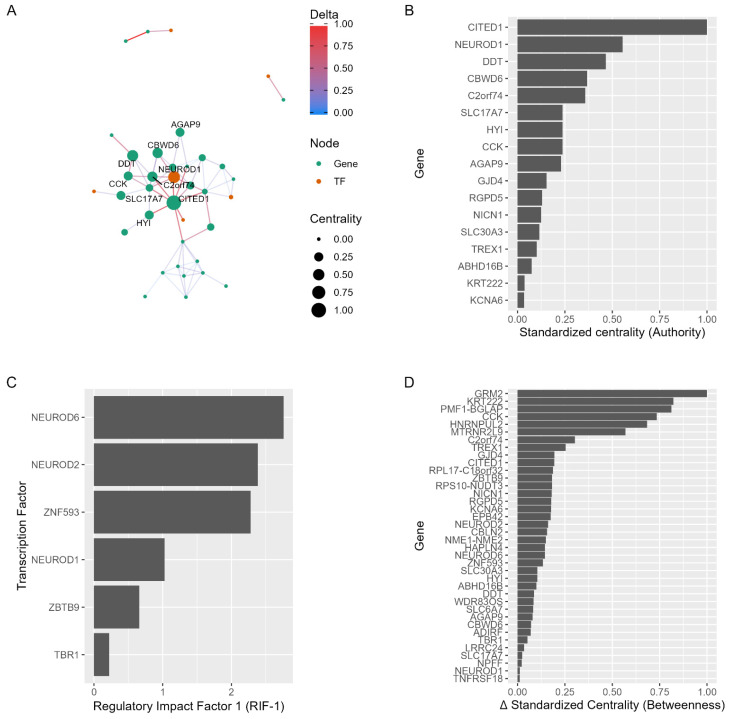
Differential network and measures of network regulators. (**A**) Differential network for PDC1 and PDC2 DEGs networks. (**B**) Bar plot for standardized centrality of genes from the differential network. (**C**) RIF 1 scores of genes from the differential network. (**D**) Difference in standardized centrality between genes from PDC1 and PDC2 networks (shown in Figure 2B,C).

A network analysis approach was used to investigate differences in gene co-expression and connectivity between the two PD clusters. Two networks were obtained using DEGs to define nodes and PCIT values to define edges between nodes. Next, the label propagation algorithm was applied on the two networks to detect communities and 2 were found in PDC1 (Figure 2B), while no distinct communities were found in PDC2 (Figure 2C). These structural differences were confirmed by the NCT test, showing that the subjects represented different PD subpopulations (*p* value < 0.0001). Communities in PDC1 were studied to identify the functional pathways enriched by their genes. Text-mining on STRINGdb showed that PDC1 community 1 (PPI score *p* value < 0.0001) was composed of DEGs belonging to Wnt/β-catenin and TGF-β/SMAD pathways, regulating adult neurogenesis [22], and functional enrichment analysis showed that DEGs in PDC1 community 1 significantly altered nuclear factor of activated T-cells 1 (*NFATc1*) transcription, which is a key regulator in Wnt signaling (TF:M04053, *p* adjusted < 0.001). To further explore functional alterations in PDC1 vs. PDC2 we decided to compare the intensity of the connections between nodes in the two networks. Although the overall level of connectivity is not different across PD subtypes (NCT, S = 34.64, *p* = 0.08), we observed 271 edges displaying different levels of strength between clusters (*p* adjusted < 0.01) (Figure 3A). Nodes in this differential network enriched the neuronal helix–loop–helix TF protein domain (PF12533, *p* adjusted < 0.01), which is required for neurogenesis.

### 2.4. Network Regulators as Drug Repurposing Candidates

DEGs from PDC1 vs. PDC2 comparison were investigated to understand their role in the disease networks, aiming to identify potential drug repurposing candidate genes. *CITED1* and *NEUROD1* were identified as the most important genes regulating the differential wiring between clusters based on the authority centrality score (Figure 3B). The influence of the bHLH TFs (*NEUROD1*, *NEUROD2*, *NEUROD6*), along with *ZNF593*, was confirmed by RIF analysis, as these DEGs were shown to be the best predictors of other DEG expression levels in the disease network (Figure 3C). To further inspect the topology of the disease networks, we computed the delta standardized betweenness centrality score between PDC1 and PDC2, identifying *GRM2*, *KRT222*, *PMF1-BGLPA*, and *CCK* DEGs as the most differentially wired between the PDC1 and PDC2 clusters (Figure 3D). The obtained set of DEGs was used in the drug repurposing pipeline.

### 2.5. Drug Repurposing Pipeline

A drug repurposing analysis was performed to identify compounds modulating selected DEGs from the PDC1 vs. PDC2 comparison (Table 1). In particular, we wanted to identify drugs showing agonist effects on the downregulated key genes driving the impaired neuroprotection network in PDC1. The obtained compounds (*n* = 42) were filtered and drugs being duplicated (*n* = 16) and inefficacious based on clinical trials (*n* = 5) were removed, along with substances showing side effects on memory or excitotoxicity (*n* = 2) and unknown modality of action (*n* = 10). The final set of drugs (*n* = 9) was mapped on *NEUROD1* and *GRM2* genes. These nine compounds displayed different modalities of action that were assessed for both preclinical and clinical evidence of their neuroprotective potential (Table 1).

### 2.6. Molecular Characterization of PDC1 or PDC2 vs. NC

**Figure 4:**
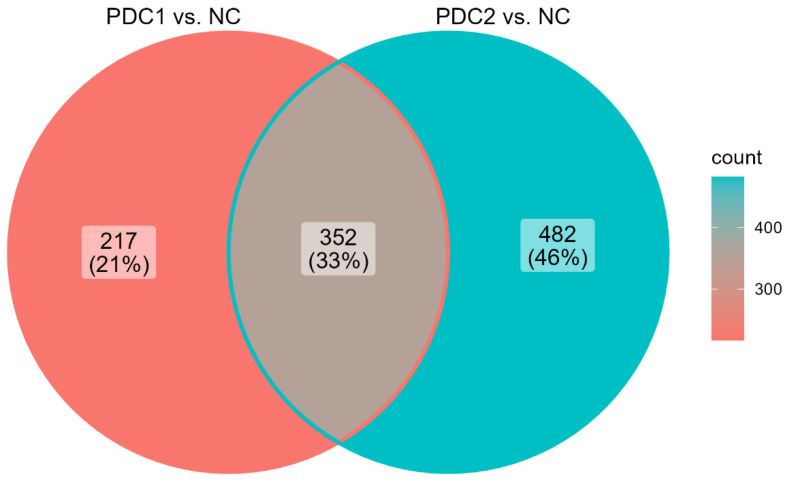
Venn diagram reporting the number of unique and overlapping DEGs found in PDC1 vs. NC and PDC2 vs. NC comparisons.

DEGs were identified for both PDC1 vs. NC and PDC2 vs. NC comparisons, aiming to assess the specific disease mechanisms of the two clusters (Figure 4). As expected, functional enrichment on PDMap enriched the entire diagram in the main map with *p* adjusted < 0.0001 for both clusters, including the Parkinsons UK Gene Ontology genes with *p* adjusted < 0.05 and *p* adjusted < 0.0001 for PDC1 and PDC2, respectively. Moreover, we studied the common DEGs resulting from each comparison to assess whether their connectivity was significantly different between PDC1 and PDC2. Two networks were built for both PDC1 and PDC2 common DEGs. Despite holding the same node structure, the networks were differentially connected (NCT, *p* < 0.0001), with 5000 differential connections showing *p* adjusted (FDR) < 0.01 (Supplementary Figure S1).

#### 2.6.1. Disease Mechanism of PDC1

**Figure 5:**
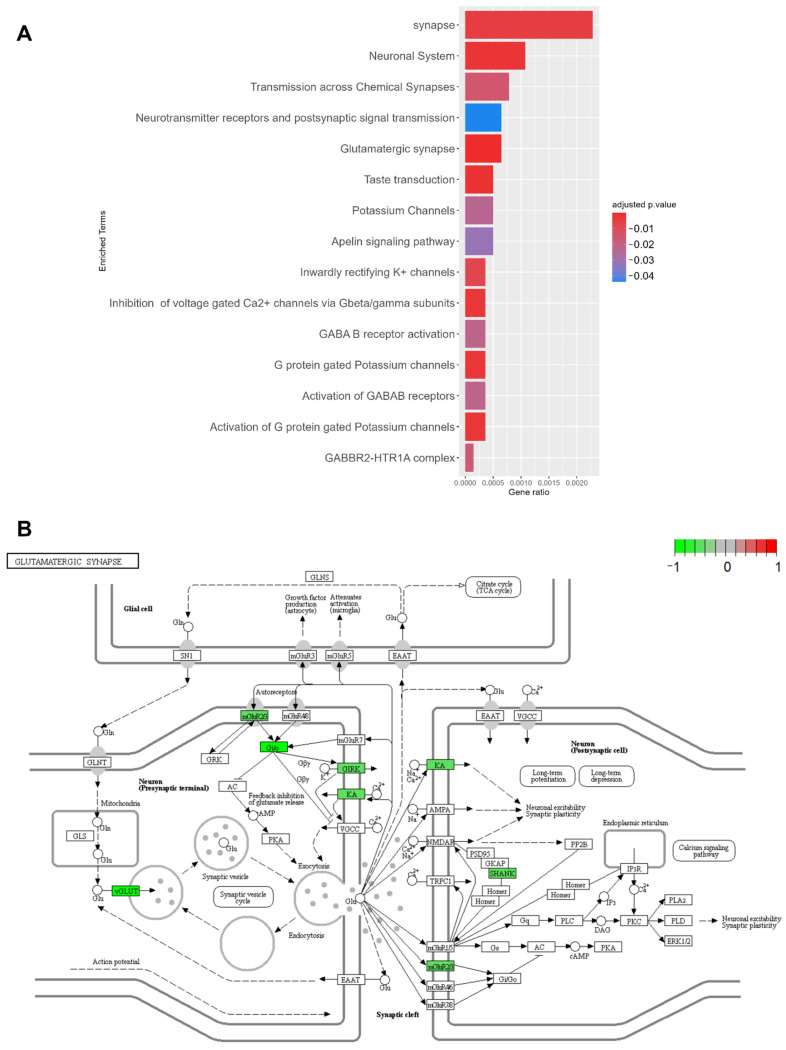
Investigation of the disease mechanism of PDC1, based on PDC1 vs. NC unique DEGs. (**A**) Enrichment analysis of DEGs shows that these genes map mostly on synaptic functions, in particular on the glutamatergic synapse. (**B**) Pheatmap illustration of the glutamatergic synapse pathway, showing downregulation in neurotransmitter uptake (vGLUT) and feedback regulation of neurotransmission (mGluR2) functions.

A total of 217 unique DEGs were identified in the PDC1 vs. NC comparison with *p* adjusted (FDR) ≤ 0.01 (Figure 4). Within this set, 5 unique TFs were found and GDA analysis revealed that 12% of these DEGs were already associated with PD. Functional enrichment analysis mapped this set of DEGs on 15 biological pathways (*p* adjusted < 0.05), mostly belonging to glutamatergic (KEGG:04724) and GABAergic signaling (CORUM:5418, REAC:R-HSA-991365, REAC:R-HSA-977444), but also including the Apelin signaling pathway (KEGG:04371) (Figure 5A) and the Sonic Hedgehog (SHH) pathway from the Parkinson’s UK Annotation Initiative (*p* adjusted < 0.05) in the PDMap (Supplementary Figure S2). The expression profiles of DEGs involved in the glutamatergic synapse were further detailed, highlighting the downregulation of several key genes underlying the glutamate balance in the synaptic cleft (Figure 5B) (*GNG13*, *SLC17A7*, *GNG11*, *GRM2*, *SLC17A6*, *GRIK1*, *KCNJ3*, *SHANK1*, *GNG3*).

#### 2.6.2. Disease Mechanism of PDC2

**Figure 6:**
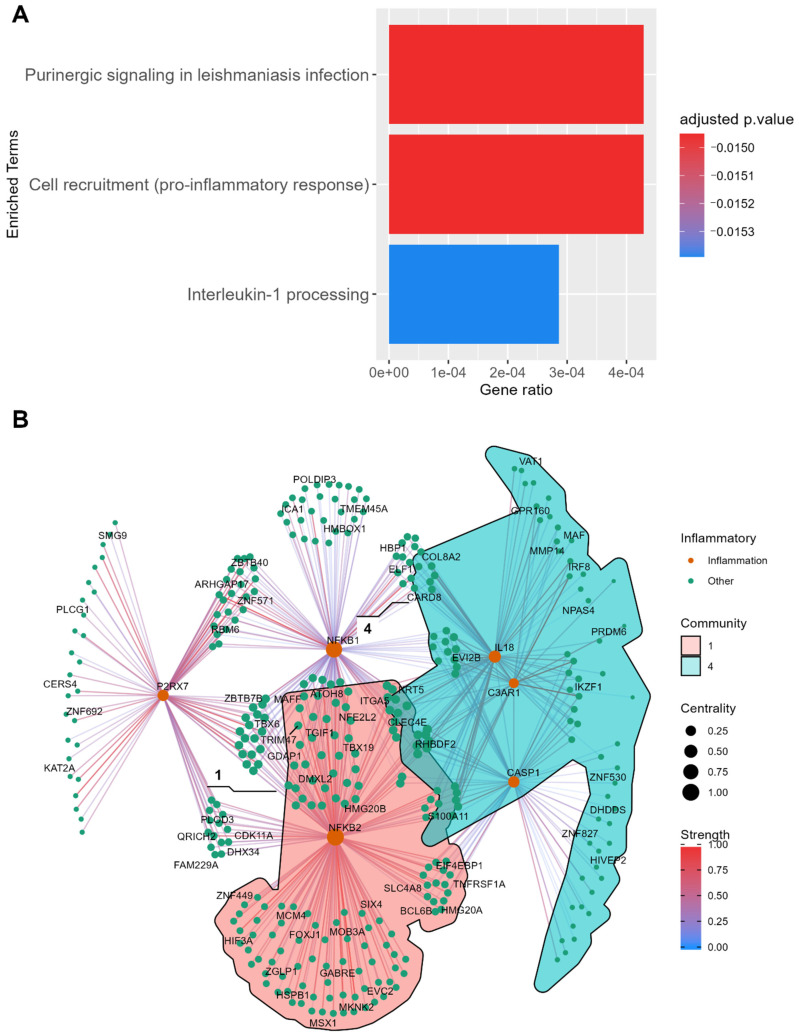
Investigation of the disease mechanism of PDC2, based on PDC2 vs. NC unique DEGs. (**A**) Enrichment analysis of DEGs shows that these genes map on inflammatory processes. (**B**) Gene network showing segregation in communities (only communities with significant PPI scores are colored). The network was built with the Kamada–Kawai layout, where each node is a gene and each edge is a PCIT value between genes.

A total of 482 unique DEGs were identified in the PDC2 vs. NC comparison, with *p* adjusted (FDR) ≤ 0.01 (Figure 4). Within this set, 2 unique TFs were found, while 10% of the DEGs were already associated with PD from GDA analysis. Functional enrichment analysis showed that PDC2 unique DEGs exclusively enriched pro-inflammatory pathways, namely cell recruitment (pro-inflammatory response), purinergic signaling in leishmaniasis infection, and Interleukin-1 processing from the REACT database (Figure 6A), as well as endoplasmic reticulum (ER) stress signaling (*p* adjusted < 0.01) and the ubiquitin proteasome system (*p* adjusted < 0.05) from the Parkinson’s UK Annotation Initiative in the PDMap (Supplementary Figure S3). These pathways were enriched by the same set of genes: *NFKB2*, *IL18*, *P2RX7*, *NFKB1*, *C3AR1*, and *CASP1*. A network analysis approach was used to further investigate this immunological subnetwork characterizing PDC2 (Figure 6B). Several communities of DEGs were found, and communities 1 and 4 reported significant PPI enrichment scores *p* values (*p* value = 0.002; *p* value < 0.0001). Community 1 significantly enriched the cell surface receptor signaling pathway (*p* value < 0.05). STRINGdb text mining on this community suggested that this set of DEGs may be involved in neuronal apoptosis mediated by oxidative stress (*CXCR4*, *4E-BP1*, *DR5*, *TNFRSF10B*, *TRAIL-R2*, *HSPB1*, *TNFRSF1A*, *BAG3*) [23]. As expected, community 4 was mainly composed of genes from the immune system (adjusted *p* value = 0.03).

## 3. Discussion

Disease subtyping is required to address PD heterogeneity in clinical manifestations and progression and improve both management strategies and research in an attempt to develop disease-modifying treatments. In this study, we proposed a hybrid workflow integrating ML, bioinformatics, and network analyses to overcome the pervasive limitations in PD subtyping, specifically addressing reproducibility, disease network identification and characterization, drug repurposing, and knowledge transferability. As a proof of concept, we analyzed RNA-Seq data from PD and NC post mortem brain samples to identify PD subtypes and to characterize the gene networks regulating common and specific disease mechanisms. In fact, we applied a data-driven approach based on network analysis and information theory and determined the most influential DEGs. Finally, we applied a drug repurposing pipeline to propose compounds holding therapeutic potential. Two PD subtypes were identified and internally validated using unsupervised ML. The obtained clusters differed by lifespan and Braak score, with PDC2 reporting a later age of death and lower neuronal depletion than PDC1. Additionally, we observed that PDC1 and PDC2 were characterized by specific disease mechanisms when compared with NC, further confirming that PDC1 and PDC2 represented two distinct subpopulations in PD. Alterations in SHH and Apelin signaling, coupled with altered glutamatergic transmission, specifically characterized PDC1, while the PDC2 unique disease fingerprint reported ER and oxidative stress mechanisms due to the increase in neuroinflammation. Most of the differences between PDC1 and PDC2 gene expression were driven by a gene network centered on the bHLH NEUROD TFs (*NEUROD1*, *NEUROD2*, *NEUROD6*) controlling cell-cycle and adult neurogenesis in the Wntβ-catenin pathway [24,25]. Genes in this community were downregulated in PDC1 vs. PDC2, suggesting an impairment of Wntβ-catenin signaling. The impairment of Wnt/β-catenin is a PD hallmark, as it regulates dopaminergic neurogenesis and survival in the subventricular zone and substantia nigra during aging [26]. Several other key genes in the Wnt/β-catenin were downregulated in PDC1, including *NFATc1* and CBP/p300 modulators (*CITED1*, *eEF1Bγ*). CBP and p300 are epigenetic factors constituting the *KAT3* family, which controls chromatin acetylation [27]. Differential CBP/p300 modulation is used to regulate nuclear receptor/Wnt/β-catenin interactions, allowing for both the maintenance of genomic integrity and neuronal plasticity during aging [28]. The present extensive network analysis revealed that *NEUROD1* and *GRM2* were the most influential genes in the network, as they were able to predict and/or influence the expression of the other DEGs. *NEUROD1* regulates the onset of neurogenesis, differentiation, and survival in the Wnt/β-catenin pathway [29,30,31], representing a key target of several applications aiming to restore dopaminergic signaling in PD [32,33,34,35,36]. In particular, *NEUROD1* chemical reprogramming has produced dopaminergic neurons from astrocytes in an in vivo PD mouse model, while its overexpression reduced the loss of the dopaminergic neurons associated with PD, thus inducing relief of symptoms [22,24,37,38]. *GRM2* encodes mGluR2 metabotropic receptors modulating neurotransmission and synaptic plasticity between the substantia nigra and subthalamic nucleus [39]. Recently, *GRM2* downregulation in the substantia nigra has been associated with PD [40] since mGluR2 is essential for the induction of long-term depression (LTD) in the substantia nigra and mGluR2 agonists have been proposed for the treatment of motor symptoms in PD [41,42,43]. Given the therapeutic potential of *NEUROD1* and *GRM2*, we used them in a drug repurposing pipeline, finding nine compounds potentially able to restore their functionality. *NEUROD1*-interacting compounds (ISX9, PD98059, deferoxamine) are currently under evaluation as disease-modifying treatments in PD [4]. ISX9 is a neural stem cell inducer aiming to enhance adult neurogenesis through *NEUROD1* overexpression [33,44,45,46]. PD98059 acts as an ERK1/2 inhibitor, counteracting the apoptotic processes harming dopaminergic neurons [45,46,47,48]. Finally, deferoxamine (DFO) is an hexadentate iron chelator approved by the Food and Drugs Administration (FDA) to be prescribed for iron and aluminum intoxication [49]. Given that free iron and aluminum deposits are known to be involved in PD pathogenesis, iron chelation may represent a promising therapeutic strategy to improve behavioral outcomes and slow down neurodegeneration [50,51]. In PD animal models, DFO chronic intranasal administration reduces motor defects and overall pathology, while it has been demonstrated that DFO treatment increases the number of neurons produced from neural stem/progenitor cells (NPCs) due to the activation of the NEUROD signaling pathway [52,53]. DFO action can also be coupled with antioxidants to counteract neurotoxicity in dopaminergic neurons of the substantia nigra, reducing oxidative stress and cellular damage [54]. Clinical trials on an orally active form of the iron chelator deferiprone (DFP) showed decreases in substantia nigra iron content resulting in improved Unified Parkinson’s Disease Rating Scale (UPDRS) scores [55]. *GRM2* modulators have risen in interest, given that allosteric modulators of G-protein-coupled receptors (GPCRs) appear to provide a new strategy to develop novel treatments in neurodegenerative diseases in general and PD in particular [43]. Moreover, GPCR positive allosteric modulators (PAMs) acting on mGluR2 can potentiate the receptor response providing higher subtype selectivity and thus reduce the activation of pathways inducing side-effects [56].

## 4. Materials and Methods

### 4.1. Systematic Data Retrieval

**Figure 7:**
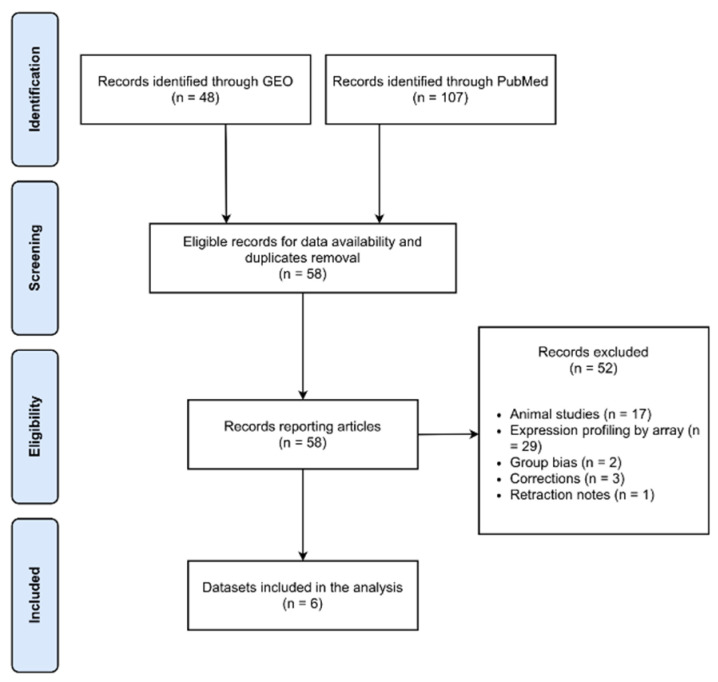
PRISMA flow diagram showing the step-by-step process of our search and selection applied during systematic data retrieval.

A comprehensive online search of the published literature in Gene Expression Omnibus (GEO) and PubMed databases (to 30 April 2021) was performed to identify all publications measuring RNA expression levels in brain tissue from idiopathic PD patients. The search strategy used a query string including as relevant keywords: “Parkinson, High throughput Sequencing”. Studies were included to retrieve data if they met the following criteria: available RNA-Seq data; sequenced RNA extracted from post-mortem human brain tissue; RNA sequenced by next-generation sequencing (NGS) technology; data must include both PD and normal control (NC) subjects’ expression matrices. Search results were reviewed by two investigators (A.T. and C.F.) who were required to agree on study selection. Any discrepancies were resolved by discussion. Preferred Reporting Items for Systematic Reviews and Meta-Analyses (PRISMA) guidelines adherence was respected to perform this systematic data retrieval [57]. After filtering, 6 datasets including transcriptomic data of 210 participants (PD = 115; NC = 94) were selected (Figure 7), namely GSE136666 [40], GSE135036 [58], GSE134390, GSE110716 [59], GSE68719 [60], and [61].

### 4.2. Raw Counts Preprocessing

Raw counts matrices were integrated by gene, and univariate anomaly detection on demographics was performed using Grubbs testing. One PD subject that proved to be an outlier for the age of death parameter was then removed. Genes with expression values below the 15th percentile were removed during the low counts filtering stage. The batch effect due to the experiment source was removed using the “ComBat-seq” function in the sva package [62,63]. “ComBat-seq” uses a negative binomial regression model to estimate batch effects based on the count matrix in RNA-seq studies. The estimated batch effect parameters are used to calculate the expected distributions if there were no batch effects in the data based on the model. Then, the “ComBat-seq” function adjusts the data by mapping the quantiles of the empirical distributions of data to the batch-free distributions [64]. Preprocessed gene counts matrices for the clustering pipeline are included in Supplementary Table S1.

### 4.3. Clustering Pipeline

After raw counts preprocessing, the clustering tendency of the PD gene-expression data was confirmed using Hopkins’ statistic (H = 0.14), while the best number of clusters (*n* = 2) was estimated using the NbClust R package [65,66]. NbClust’s approach to clustering validity is based on the relative criteria, which consists of the evaluation of a clustering structure by comparing it with other clustering schemes resulting from the same algorithm but with different parameter values [66]. The clustering pipeline included 7 algorithms from the factoextra R package [67] (fuzzy clustering; k-means, hierarchical_kmeans; clara, agnes; pam; hclust; diana) over several distance metrics and measures (Supplementary Table S2) and the final clustering method was selected based on the maximum average Silhouette width and Dunn index methods (Supplementary Table S2). The silhouette (S) and Dunn (D) coefficients measure how similar an observation is to its own cluster (cohesion) compared to other clusters (separation), which indicates how well an observation was classified [68]. Since S and D coefficients can be computed with any distance metric, they can be compared across multiple models. Moreover, it was demonstrated that S and D coefficients are among the most reliable internal validation metrics in five different aspects: monotonicity, noise, density, subclusters, and skewed distributions [69].

### 4.4. Statistical Analyses

All the statistical analyses were performed in R v. 4.1.0 [70]. A seed was set to 12345 for all algorithms needing pseudorandomization. A Wilcoxon rank-sum test was performed to assess differences in age of death in PDC1 vs. PDC2 comparison [71], whereas Fisher tests were used to evaluate differences in Braak score and sex in the same comparison [72].

### 4.5. Differential Expression Analysis

Differential expression analysis was performed using limma for each comparison and gene expression matrices were transformed in log_2_ counts per million (CPM) before modeling [73]. Finally, moderated t-statistic and F-statistic along with log-odds of differential expression were computed by empirical Bayes moderation of the standard errors. Only genes with *p*-value adjusted for false discovery rate (FDR) ≤ 0.01 were considered differentially expressed genes (DEGs) [74]. Over-representation analysis (ORA) was performed to map genes on their functional pathways. DEGs were queried on all gene ontology (GO) domains: biological processes (BP), cellular components (CCs) and molecular functions (MFs). Similarly, DEGs were mapped on the CORUM database, which provides manually annotated protein complexes, and the Kyoto Encyclopedia of Genes and Genomes (KEGG) database using g: Profiler API [75]. Moreover, a gene-disease association (GDA) analysis was performed using DisGeNET to retrieve DEGs associated with PD (tag:C0030567) [76]. Unique DEGs from PDC1 and PDC2 vs. NC comparisons were mapped on the Parkinson’s Disease Map (PDMap), including the Parkinson’s UK Annotation Initiative, to identify their specific disease pathways [77]. The obtained pathways were considered as differentially modulated with *p* adjusted ≤ 0.05 (FDR) and KEGG pathways were visualized using the pathview package [78]. Critical TFs among DEGs were identified using the CeTF package, and regulatory impact factor (RIF) scores were computed for each of the identified TFs [79]. Complete results from the differential expression analysis are reported in Supplementary Tables S3 and S4 for PDC1 vs. PDC2 and PDC1/2 vs. NC, respectively.

### 4.6. Gene Network Analysis

The gene networks in PDC1 and PDC2, as well as the differential network between the clusters, were investigated using the partial correlation coefficient with information theory (PCIT) algorithm to estimate connectivity among nodes. PCIT identifies meaningful correlations to define edges in a weighted gene co-expression network [79]. Importantly, PCIT allows one to draw a co-expression gene network while controlling the moderation effect of other genes. For example, for every trio of genes in x, y, and z, the partial correlation coefficient between x and y given z indicates the strength of the linear relationship between x and y that is independent of (uncorrelated with) z. Statistical significance of the association between x and y is evaluated by computing the tolerance level to be used as the local threshold for capturing significant associations for every trio of genes. The tolerance level here is defined as the average ratio of partial to direct correlation [80]. Finally, each network was optimized by pruning isolated nodes and looped or multiple edges. The obtained networks were represented using the force-directed Kamada–Kawai algorithm to assure that geometric distances between vertices closely corresponded to the underlying graph distances. Moreover, distinct communities of genes in PDC1 and PDC2 networks were identified using the label propagation algorithm from the tidygraph package, while weights of genes in the networks were estimated using the betweenness centrality score from the same package [81]. The functions of the identified communities and their protein–protein interaction (PPI) enrichment scores were investigated using the protein–protein interaction network from the STRINGdb package [82]. The networks were compared to identify both structural differences and changes in the overall level of connectivity using the NetworkComparisonTest (NCT) package [83]. To build the differential network in PDC1 vs. PDC2 comparison, we tested the edge strength invariance hypothesis, meaning that a specific edge is identical across subpopulations. The edges and nodes showing differential strength levels were used to represent the differential network between the two clusters, while the authority centrality measure was computed to estimate the weight of the nodes in the network [81].

### 4.7. Drug Repurposing

The DEGs from PDC1 vs. PDC2 comparison were used in a drug repurposing pipeline. We selected as input the top-5 most influential DEGs in the gene network based on authority, RIF1, RIF2, and betweenness scores. The selected DEGs were queried on the Drug Gene Interaction Database (DGIdb) and the Drug Repurposing Hub from the Broad Institute to obtain their interacting compounds and modality of action. The Drug Repurposing Hub is a curated and annotated collection of FDA-approved drugs, clinical trial drugs, and pre-clinical tool compounds, while DGIdb is a data mining platform exploring the druggable genome for personalized medicine [84,85]. The DGIdb mining algorithm explores over 30 trusted sources, including scientific literature and curated databases [84]. The obtained set of drugs was filtered with the following criteria: duplicated, inefficacious, side effects on memory or excitotoxicity, and unknown modality of action. The final set of drugs was then evaluated based on their target and modality of action [84,85]. Each compound’s literature data was filtered to include only preclinical experiments or concluded clinical trials on neurodegenerative diseases. Finally, data from ClinicalTrials.gov (accessed on 10 May 2021) were retrieved to verify ongoing clinical trials on neurodegenerative diseases involving the administration of the identified compounds [86].

## 5. Conclusions

Hybrid workflows combining network biology and artificial intelligence have the potential to discover novel mechanisms and promising drug targets for complex diseases [21]. Despite this potential, these techniques have rarely been combined in a data-driven pipeline. We implemented a fully data-driven pipeline for PD subtyping, aiming to gather knowledge for precision medicine applications. Collectively, our results point out that two clusters representing distinct PD subpopulations can be identified from RNA-Seq data. Adopting a rigorous data-driven pipeline for disease subtyping allowed us to stratify homogeneous groups of individuals based on their unique pattern of gene expression. Interestingly, PD subjects belonging to PDC1 and PDC2 were consistently different in the Wnt/β-Catenin signaling pathway, which regulates neuronal survival, adult neurogenesis, and plasticity. However, when compared to NC, PDC1 and PDC2 differed also by disease mechanisms. PDC1 was characterized by an altered glutamatergic transmission, while PDC2 was characterized by altered inflammatory pathways. These results were independently validated on the PDMAP from the University of Luxembourg, which is a manually curated knowledge repository established to describe molecular mechanisms of PD. These alterations in neuroprotection and distinct disease machinery were reflected on clinical data, as PDC1 and PDC2 were characterized by different ages at death and Braak scores. Using a data-driven methodology for subtype identification and characterization could result in the identification of disease-modifying treatments specifically suited for one subtype. To this extent, we performed a drug-repurposing pipeline using as input the key genes regulating the alteration in expression profiles of PDC1 and PDC2, identifying nine drugs at various stages of FDA approval. This set of compounds shared a known or predicted neuroprotective effect. This workflow is in line with the aims of precision medicine, for which detecting disease subtypes enhances diagnostic precision, finally helping to determine specific treatments for well-defined patients [87]. Although researchers are using similar in silico approaches to unravel the regulatory pathways underneath complex diseases and to identify key druggable disease networks [20,21,88], reduced data availability represents a major limitation in this workflow, as pivotal clinical information like age of PD onset, treatment type and duration, or comorbidities is often lacking in public repositories. The lack of clinical characteristics in in silico unsupervised analyses prevents this kind of application from being translated into clinical practice in a timely manner. Moreover, limitations in data availability have hindered knowledge translation from the brain to more easily available tissues in clinical practice, such as blood samples. Thus, the translation of the findings into biological understanding remains a major challenge to be addressed in future research. As the field moves forward, better data practices are needed to keep up with the increasing availability of novel technologies and the need to implement artificial intelligence tools for biomedical purposes [89]. Here, we showed how transcriptomic data and unsupervised ML can be leveraged to identify and characterize distinct subpopulations of idiopathic PD while proposing tailored potential treatments, enabling a precision medicine approach to complex neurodegenerative diseases.

## Figures and Tables

**Table 1:** Compounds and drugs obtained from the drug repurposing pipeline. The modality of action of each compound was identified on DGIdb and validated through extensive literature assessment; PMIDs are reported in the table.

Gene	Drug	ChEMBL-ID	Phase	Modality of Action	PMID
*NEUROD1*	PD98059	CHEMBL35482	Preclinical	ERK1⁄2 pathway inhibitor	12297313
					28337120
					30274251
					16787571
*NEUROD1*	DEFEROXAMINE	CHEMBL556	Launched	hexadentate iron chelator	16697980
					32926630
					23531432
					22754573
					31868679
					33513737
					33805195
*NEUROD1*	ISX9	CHEMBL1222381	Preclinical	neural stem cell inducer	29311646
					18552832
					26407349
					28656155
					22542682
					28216149
*GRM2*	JNJ-40411813	CHEMBL3337527	Phase 2	glutamate receptor positive allosteric modulator	25462291
					25586401
					25735992
*GRM2*	LY2979165	CHEMBL3544939	Phase 2	glutamate receptor positive allosteric modulator	32052375
					33071070
					29564482
*GRM2*	LY2969822	CHEMBL3545270	Phase 1	glutamate receptor agonist	28177520
					31306647
					30934533
*GRM2*	LY404039	CHEMBL375611	Phase 1	glutamate receptor agonist	32403118
					32403118
*GRM2*	BINA	CHEMBL593013	Preclinical	glutamate receptor positive allosteric modulator	16046122
					16608916
					17526600
					24076101
					28472649
*GRM2*	CBiPES	CHEMBL4303163	Preclinical	glutamate receptor positive allosteric modulator	15717213
					19951716
					22659090

## Data Availability

Original data are available from the NCBI Gene Expression Omnibus at https://www.ncbi.nlm.nih.gov/geo/ (accessed on 19 February 2022). The preprocessed data table, ready for the unsupervised machine learning pipeline, is available in the Supplementary Materials.

## Supplementary Materials

The following supporting information can be downloaded at: https://www.mdpi.com/article/10.3390/ijms23052557/s1.
