# Supplementary figures and images for: A Hybrid Machine Learning and Network Analysis Approach Reveals Two Parkinson’s Disease Subtypes from 115 RNA-Seq Post-Mortem Brain Samples
Source: Int J Mol Sci. 2022 Feb 25;23(5):2557. doi: 10.3390/ijms23052557 (PMC8910747; doi:10.3390/ijms23052557)

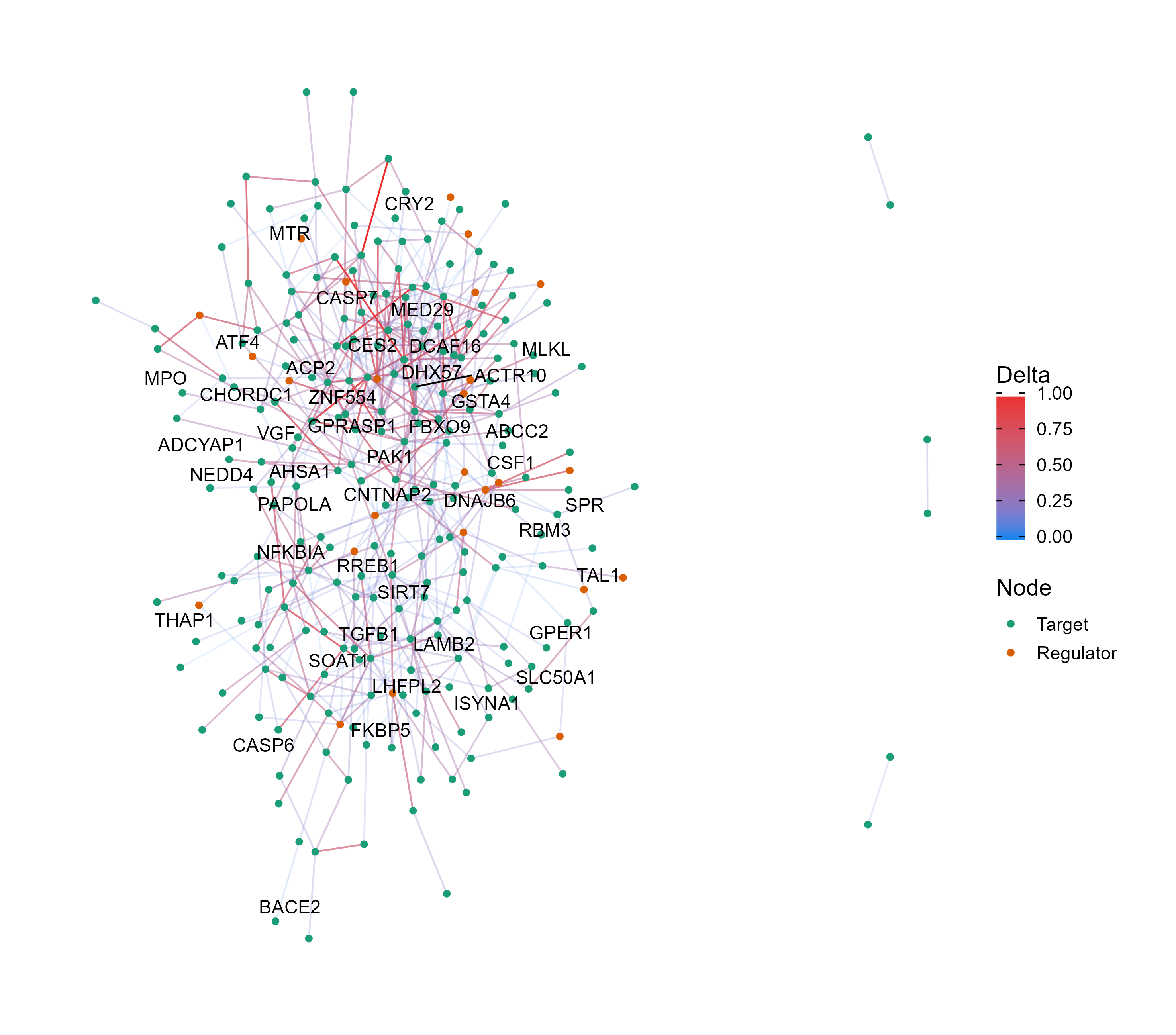

Supplement: Supplementary file 1 [file ijms-23-02557-s001.zip › S1.png]

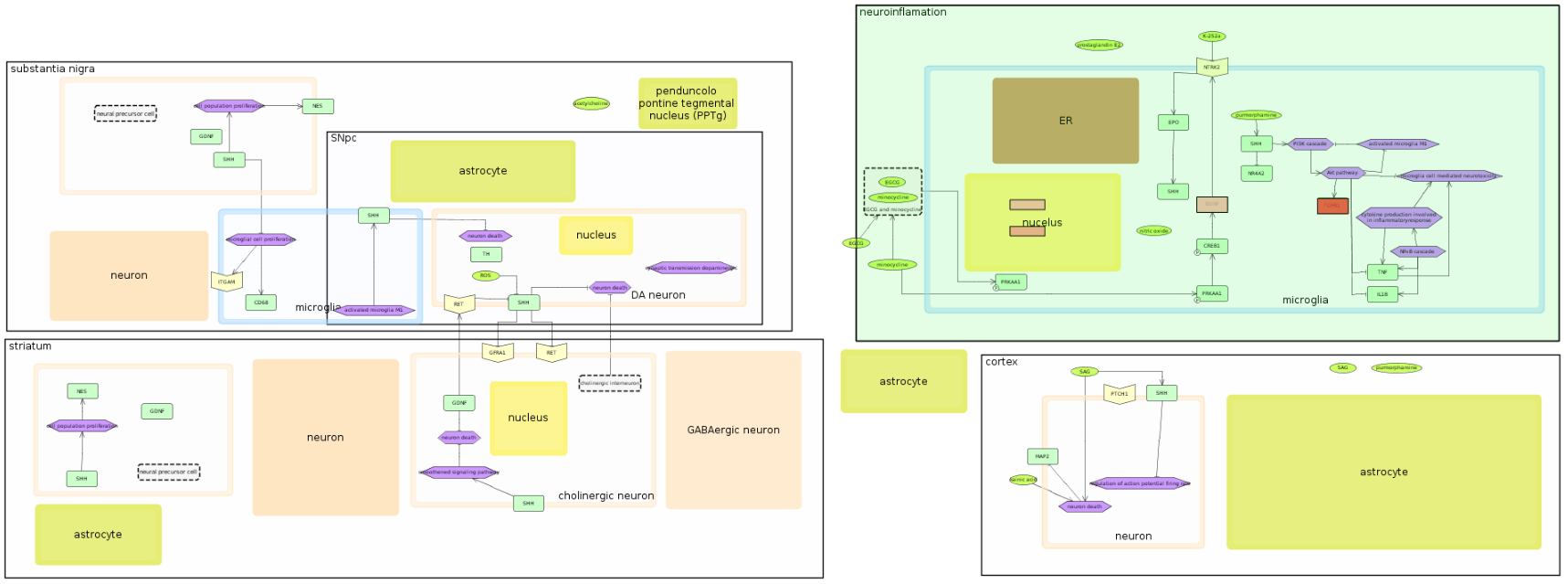

Supplement: Supplementary file 1 [file ijms-23-02557-s001.zip › S2.png]

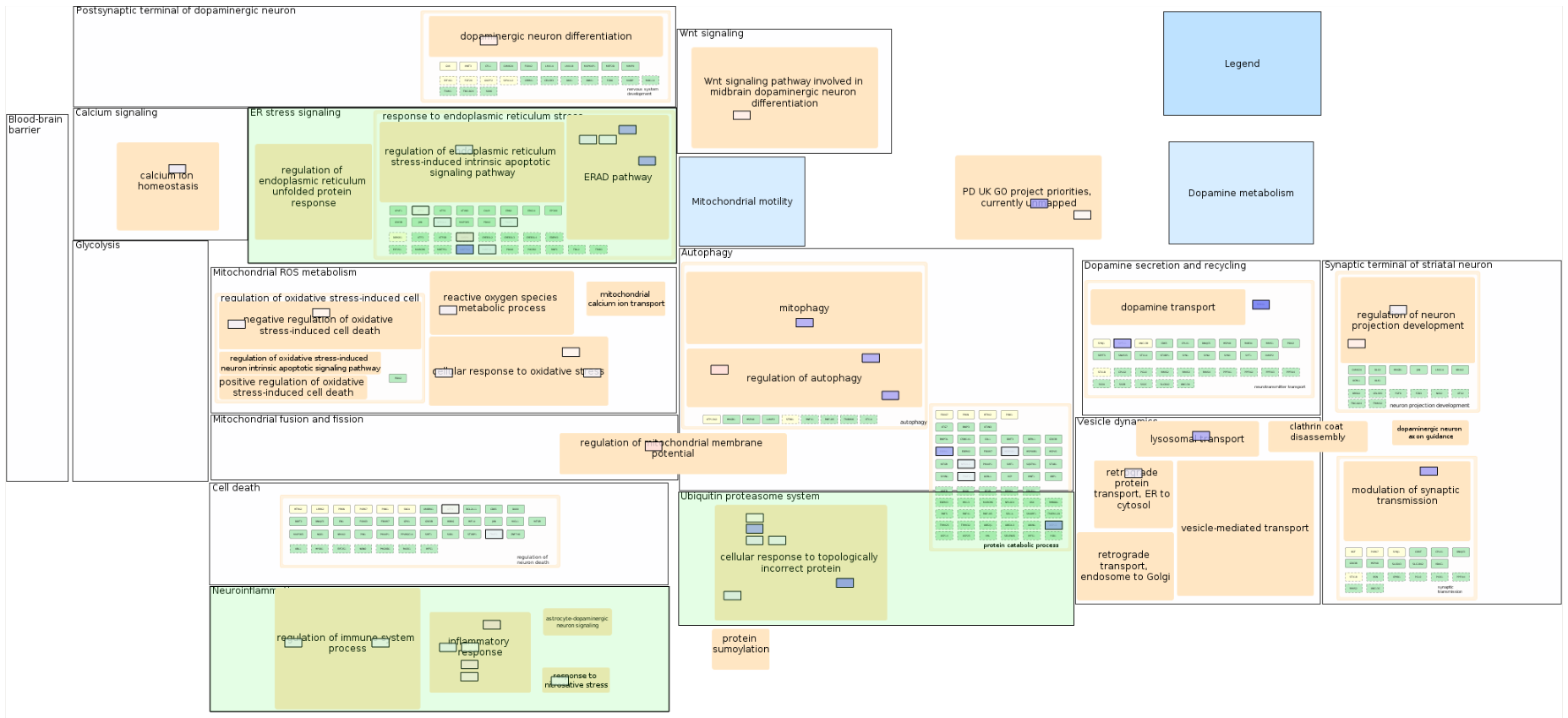

Supplement: Supplementary file 1 [file ijms-23-02557-s001.zip › S3.png]
